# The influence of the subcortex and brain stem on overeating: How advances in functional neuroimaging can be applied to expand neurobiological models to beyond the cortex

**DOI:** 10.1007/s11154-022-09720-1

**Published:** 2022-04-05

**Authors:** Po-Han Kung, Carles Soriano-Mas, Trevor Steward

**Affiliations:** 1 Melbourne School of Psychological Sciences, Faculty of Medicine, Dentistry and Health Sciences, University of Melbourne, Parkville, VIC Australia; 2 Melbourne Neuropsychiatry Centre, Department of Psychiatry, The University of Melbourne, Victoria, Australia; 3 Psychiatry and Mental Health Group, Institut d’Investigació Biomèdica de Bellvitge (IDIBELL), Neuroscience Program, L’Hospitalet de Llobregat, Spain; 4 CIBERSAM, Carlos III Health Institute, Madrid, Spain; 5 Department of Social Psychology and Quantitative Psychology, University of Barcelona, Barcelona, Spain

**Keywords:** Obesity, Binge eating, Neuroimaging, fMRI, Subcortex, Thalamus

## Abstract

Functional neuroimaging has become a widely used tool in obesity and eating disorder research to explore the alterations in neurobiology that underlie overeating and binge eating behaviors. Current and traditional neurobiological models underscore the importance of impairments in brain systems supporting reward, cognitive control, attention, and emotion regulation as primary drivers for overeating. Due to the technical limitations of standard field strength functional magnetic resonance imaging (fMRI) scanners, human neuroimaging research to date has focused largely on cortical and basal ganglia effects on appetitive behaviors. The present review draws on animal and human research to highlight how neural signaling encoding energy regulation, reward-learning, and habit formation converge on hypothalamic, brainstem, thalamic, and striatal regions to contribute to overeating in humans. We also consider the role of regions such as the mediodorsal thalamus, ventral striatum, lateral hypothalamus and locus coeruleus in supporting habit formation, inhibitory control of food craving, and attentional biases. Through these discussions, we present proposals on how the neurobiology underlying these processes could be examined using functional neuroimaging and highlight how ultra-high field 7-Tesla (7 T) fMRI may be leveraged to elucidate the potential functional alterations in subcortical networks. Focus is given to how interactions of these regions with peripheral endocannabinoids and neuropeptides, such as orexin, could be explored. Technical and methodological aspects regarding the use of ultra-high field 7 T fMRI to study eating behaviors are also reviewed.

## Introduction

Worldwide obesity prevalence has reached historically unsurpassed levels, with a 2018 World Health Organization report identifying 39% of adults as being overweight, and 13% having obesity [1]. Lifestyle interventions focused on modifying eating behaviors and increasing physical activity are generally ineffective in providing a long-term maintenance of weight loss [2]. As such, comprehensively understanding the neurobiological drivers of overeating - a primary factor responsible for increases in energy intake [3] − is a fundamental area of obesity research. Although overeating does not always occur within the context of a binge eating episode, there is evidence supporting that binge eating and obesity co-occurring is especially problematic as it is associated with more frequent weight fluctuations, less exercise, increased calorie consumption, and more severe psychopathology [4, 5]. Likewise, stress and negative affect are commonly reported to precipitate both overeating and binge eating episodes, suggesting that the relationship between negative affect and overeating is not unique to individuals with clinical eating disorders [6].

Functional magnetic resonance imaging (fMRI) has proven to be particularly insightful in elucidating the neural mechanisms related to the pathophysiology underlying overeating in humans [7]. fMRI measures changes in local blood oxygen level dependent (BOLD) signal to generate a brain map corresponding to blood flow to active neurons, which permits, for example, the comparison of brain activity in groups of healthy weight controls and individuals with obesity. fMRI studies examining neural response in overeating and obesity have merged in identifying irregularities in neural systems implicated in emotion regulation, reward seeking, sensory processing, and cognitive control [8–12]. Most commonly, the neuroimaging literature posits that maladaptive feeding patterns result from alterations in reward systems that regulate hedonic feeding [13–15]. According to this viewpoint, the appetitive sensory properties of highly palatable foods override signals from physiological negative feedback systems that are in place to restrain overeating. This position is supported by evidence demonstrating that individuals with obesity have fewer dopamine D2 receptors in the striatum than lean individuals and display reduced response in regions critical for reward processing when consuming palatable food [16]. This selectivity in reward response has been suggested to parallel with the phenomenon of tolerance seen in drug addiction [17]. In addition, palatable foods and their predictive cues may be afforded with increased incentive salience underpinning the *wanting* of food, which motivates approach behaviors towards food-related rewards. Neurobiologically, the generation of food reward wanting involves increased activity in an extensive network of mesocorticolimbic structures, such as the dorsal striatum (DS), ventral tegmental area (VTA), nucleus accumbens (NAcc), lateral hypothalamus (LH) and the central nucleus of the amygdala, which modulate multiple neurotransmitter systems (e.g., dopamine, endocannabinoid, glutamate) to incorporate hedonic, learning, emotive, and homeostatic signals in order to incite cravings [18].

Cravings for food are understood to interfere with competing cognitive demands as some individuals with obesity may be biased to automatically direct attentional resources to craving-related cues [19, 20]. This is further supported by growing research demonstrating that addictive-like eating and obesity are associated with impaired executive functioning and inhibitory control [21–25]. Ultra-processed foods and drinks in particular have been linked to potentially impacting serotonergic and dopaminergic neurotransmission and leading to addiction-like behaviors stemming from alterations in inhibitory processes mediated by the prefrontal cortex [26, 27]. However, the prominence of the conscious perception of the hedonic sensory properties of highly palatable foods in driving overeating has come into question as there exists little empirical support for the notion that the heightened liking of food contributes to obesity or is endorsed by individuals with obesity [28–30]. This suggests that the choice to eat unhealthy foods is not necessarily due to increased liking for these foods, but instead raises the possibility that alterations in subcortical body-to-brain neural pathways linking gastrointestinal nutrient sensors to brain regions mediating reward may contribute to a dissociation between value-based brain responses and internal satiety signals.

Subcortical and brain stem regions are more difficult to access than the cortex with MRI due to their smaller size and greater distance from radio frequency coils. However, major neuroimaging advances now provide the improved signal and the resolution required to accurately examine subcortical and brainstem function [31, 32]. The higher magnetic field strengths of 7-Tesla (7 T) MRI allow for the development of research questions that leverage the increased signal-to-noise ratio and improved sensitivity to task-elicited changes in BOLD signals [33]. Enhanced signal sensitivity provides higher temporal and spatial resolution necessary to observe neural activities in smaller brain structures (e.g., subcortical nuclei). Furthermore, improved BOLD contrasts observed using 7 T scanners may yield enhanced statistical power, enabling more robust inferences on both the individual and group level [34, 35].

Here, we seek to expand current neurobiological models of overeating by integrating the function of thalamic nuclei, the brainstem and subdivisions of the basal ganglia. Although substantial technical considerations for imaging the subcortex remain unresolved, we provide recommendations and considerations for mapping these regions when studying the neural mechanisms underlying excess food intake. It is worth noting that the drivers of food intake cannot be wholly grasped without bearing in mind the contributions of the brain–gut axis – an interdependent system impacting brain function and eating behavior via biochemical signaling between the endocrine and nervous system through hormonal peptides in the gastrointestinal tract. Given that several comprehensive reviews on this topic have recently been published [36–38], we will primarily focus on how functional neuroimaging techniques may be harnessed to address gaps in the field’s knowledge of how subcortical and brainstem structures contribute to overeating.

## Lateral hypothalamus

The hypothalamus is recognized as an essential interface of homeostatic energy regulation. It consists of numerous subdivisions that interact to coordinate the regulation of body weight, body temperature, feeding and autonomic arousal [39]. In particular, the lateral hypothalamus (LH) is positioned on the forebrain-brainstem axis to integrate motivational signals from the corticostriatal circuit with homeostatic information from the brainstem to modulate eating behaviors [40]. Given that there already exist multiple extensive reviews on the role of the LH in eating behaviors [40–42], this section will aim to only provide a brief summary on LH function and focus on other, less extensively studied subcortical regions for the remainder of the review.

The LH receives innervation from the parabrachial nucleus and periaqueductal gray, and projects towards downstream dopaminergic systems, including the ventral tegmental area (VTA) and the nucleus accumbens (NAcc). In addition, neurons in the LH are sensitive to peripheral nutritional hormones (e.g., insulin, leptin, ghrelin) and release influential neuropeptides, such as melanin-concentrating hormones and orexin, to affect dopamine release in the VTA and NAcc [43, 44]. Behaviorally, this allows for the dynamic adjustment of food-related reward value, as well as for changes in motivation to approach food rewards based on the organism’s homeostatic state. Optogenetic activation of the melanin-concentrating hormone neurons in the LH during the ingestion of an artificial sweetener sucralose results in increased striatal dopamine release and an enhanced preference for sucralose [45]. Moreover, food-cue induced ghrelin has been found to engage the ventral hippocampus and its efferent LH orexin-expressing neurons to mediate conditioned feeding behaviors, while the administration of orexin receptor antagonists weakened cue-induced feeding in sated rats [46, 47]. Recent findings also implicated the LH in the acquisition and memory storage of food-related associative learning [48]. Together, evidence clearly demonstrates the LH as a fundamental player in the cognitive, motivational, and homeostatic processes relevant to the onset of eating behaviors. With that said, it remains unclear the extent to which dysregulated crosstalk between the LH and cortical, thalamic, and brainstem regions underpins disordered eating in humans. Future human neuroimaging studies could investigate how network dysfunctions involving the LH may give rise to behavioral urges to eat in the absence of hunger.

## Thalamus

### Paraventricular thalamus

The paraventricular thalamus (PVT) is a midline thalamic nucleus traditionally associated with arousal and fear-processing. Increasing evidence from animal studies suggests that the PVT may function as an interface between the homeostatic and hedonic systems that drive eating behaviors [49–52]. The PVT receives input from the brainstem regions (e.g., nucleus of the solitary tract, parabrachial nucleus) [53, 54], and hypothalamic nuclei which integrate nutritional signals to regulate energy expenditure and food consumption [55, 56]. Specifically, the PVT receives projections from the LH [40]. Furthermore, rodent studies have highlighted the extensive anatomical connections that the PVT shares with regions involved in reward learning [50], including afferent innervations from the medial prefrontal cortex (MPFC) [57] towards the NAcc [51].

The MPFC-PVT pathway has been found to be sensitive to reward-predictive cues and is suggested to influence appetitive behaviors via modulatory effects on reward learning. Optogenetic activation of neurons connecting the dorsomedial PFC and the PVT has been found to suppress anticipatory licking to conditioned reward-predictive cues [57]. This contrasts with brainstem and hypothalamic inputs to the PVT, which are primarily associated with the communication of viscerosensory and nutritional information that incorporate whole-body energy status and shape motivational processing [52, 58]. Moreover, recent studies identified neurons in the PVT that are sensitive to circulating glucose levels and that project to the NAcc [59, 60]. Functionally, hypoglycemia has been shown to activate the PVT-NAcc neurons expressing glucose transporter Glut2 to encourage sucrose seeking in mice, while glucoprivation has been demonstrated to induce feeding behaviors controlled by the pathway between the ventrolateral medulla and the NAcc via the posterior PVT. However, it is not yet clearly understood how these systems converge on the human PVT to influence eating behavior. Functional neuroimaging techniques could be harnessed to examine potential differences in glucose-induced activation of the PVT-NAcc pathway in individuals who overeat versus those who do not.

Beyond hedonic and homeostatic eating, the PVT has also been implicated in the modulation of ingestive behaviors under stress. The PVT receives stress and arousal-related information via the transmission of orexin and melanin-concentrating neuropeptides from the brainstem and the LH [49, 56]. While the PVT-LH pathway may be associated with general arousal and approach behaviors, the interconnections between the PVT, MPFC, amygdala, and the hippocampal formation have been associated with fear memory acquisition and retrieval [52, 61, 62]. The PVT modulates pathways between the central nucleus of the amygdala (CeA) and the NAcc and has been implicated in the regulation of stress and negative emotional behaviors [63]. In the context of eating behaviors, the PVT-CeA pathway has been suggested to affect taste perception, and to facilitate the inhibition of eating under stressful conditions [64, 65]. Functional neuroimaging holds the potential to assess how these subcortical networks contribute to human overeating behaviors when facing aversive stimuli, with paradigms exploring response to negative affect and food withdrawal potentially serving as a proxy measure of emotional eating.

### Mediodorsal Thalamus

Higher-order cognitive processes, such as decision making and abstract reasoning, have been increasingly attributed to the mediodorsal thalamus (MD), a group of thalamic nuclei which project extensively throughout the PFC [66]. In contrast to other thalamic nuclei associated with sensory and motor processing, the MD is anatomically defined by its driving afferents from the PFC, while exhibiting minimal connectivity with sensory or motor circuits. Interestingly, MD lesions frequently produce cognitive impairments that resemble those observed following prefrontal lesions [67], indicating that cognitive control cannot be fully understood without probing the reciprocal thalamocortical circuitry that mediates it [68]. Moreover, recent research using 7 T fMRI has found evidence to support the MD having a causal excitatory influence on PFC regions during complex processes such as self-directed thought and cognitive restructuring [69, 70].

Although primarily associated with cognitive functioning and not forming part of the canonical taste pathway, there is evidence supporting a role for the MD in influencing food intake. For instance, infarcts of the MD have been found to negatively impact with hedonic perception of food, with odors and taste being perceived as either neutral or unpleasant [71, 72]. One recent study found strong evidence to support the presence of an amygdala–thalamic circuit acting as a central gain mechanism for taste perceptions [65]. Here, dynamic causal modelling revealed that the connection strength between outputs from the central nucleus of the amygdala to the MD and ventral posterior thalamus predicted individual response differences to taste. This implies that the MD may form part of a tripartite circuit, along with the amygdala and posterior orbitofrontal cortex (OFC), to shape the salient and physiological meaning of afferent sensory information [73]. This framework aligns with studies in nonhuman primates demonstrating MD projection to the posterior OFC – a major site of taste and smell information convergence [74]. However, it remains unclear whether the neural circuitry underlying central gain mechanism is altered in individuals with a tendency to overeat or how this pathway is modulated under fasting and satiated states.

By virtue of receiving dual cortico-thalamic inputs from pyramidal neurons in layer V and modulatory inputs in layer VI of the PFC, the MD has been posited to serve as a relay for higher-order functions mediated by the PFC [66]. For instance, recent research has demonstrated strong excitatory effects from the MD to sustain local PFC connectivity and to enable neural sequences to emerge that maintain attentional control [75]. In the context of gating food cues, the MD-cortical communication may be especially important in learning stimulus-reward associations [76]. Importantly, pharmacological inactivation of the MD has revealed that MD is required for food reward valuation and action selection–processes which are supported individually by the amygdala and the orbitofrontal cortex, respectively [77–79]. These findings suggest that the MD may therefore play a key role in updating the value of food reward and in selecting goal-directed behavior. Considering that deficits in behavioral flexibility and attentional control are characteristic of addictive-like food intake behavior [80, 81], it would be of interest to examine the extent to which alterations in thalamo-cortical networks are associated with compulsive eating patterns.

## Brainstem

### Ventral tegmental area

The ventral tegmental area (VTA) is commonly understood to act as a central node in the dopaminergic system and has been extensively studied in the context of motivational and addictive behaviors [82]. More specifically, the VTA is recognized to contribute to food valuation by adjusting reward signals and modulating stress response pathways via the projections it receives from the LH and by modulating dopamine signaling in the NAcc [39, 83–85]. Current neurobiological models of ingestive behaviors posit that the LH may transmit information regarding energy expenditure and intake by modulating VTA activity via neuropeptides, such as neuropeptide Y and orexin, in order to influence decision-making and hedonic eating signals encoded in the dopamine system [84]. Furthermore, the VTA is also sensitive to peripheral appetitive hormone levels (e.g., insulin, ghrelin, leptin), and has been suggested to affect food seeking behaviors via the adjustment of food reward value [86]. Notably, the exposure to stress may mediate feeding via the effect of endocannabinoids and stress hormones (e.g., corticotropin-releasing factor), or by modulating the influence of appetitive hormones on the VTA [84, 87]. In mice, endocannabinoids have been shown to promote the hedonic value of palatable food by increasing dopaminergic release from the VTA to the NAcc [88]. While insulin in the VTA has been reported to suppress preference and anticipatory behaviors to food-related cues in mice, this inhibitory effect was contingent on endocannabinoid-mediated inhibition on presynaptic glutamate release [89]. However, a recent human fMRI study reported a non-significant association between peripheral endocannabinoid concentrations and functional connectivity between the VTA and the LH, despite significant correlations between endocannabinoid levels and the functional connectivity between key regions of the brain’s reward, salience, and homeostatic networks [90]. Future fMRI studies should leverage a larger sample size and ultra-high resolution imaging to further explore how the VTA may be implicated in eating behaviors moderated by the endocannabinoid system.

Relatedly, decreased glucose levels in food-restricted mice has been shown to activate glutamate and orexin co-expressing neurons in the LH to excite dopaminergic neurons in the VTA [91]. Direct manipulations of the LH-VTA pathway in rodent models have been shown to modulate eating behaviors, with photoactivation of LH GABAergic projections to the VTA inducing appetitive and consummatory behaviors in food-restricted mice [92], and low-frequency optogenetic stimulation of the same pathway eliciting increased eating in food-sated mice [93]. Interestingly, the inhibition of the LH-VTA pathway reduced compulsive sugar consumption, but not homeostasis-driven feeding in hungry mice, potentially providing a target for treating compulsive overeating [94]. In humans, glucose intravenous infusion has been shown to attenuate VTA activation preference to high- compared to low-calorie food cues [95], and glucose ingestion has been observed to contribute to a decrease of fMRI signal in the hypothalamus of normal weight individuals, but not individuals with obesity [96, 97]. Whether prolonged hypothalamic activity in individuals with obesity, putatively indicating an insensitivity to increased glucose level, may promote compulsive overeating via the sustained excitatory effect of the LH on the VTA remains a hypothesis that may be of interest for future functional neuroimaging studies.

### Locus coeruleus

The locus coeruleus (LC) is a small nucleus positioned deep in the dorsal pons within the brainstem with broad and divergent axonal pathways that provide norepinephrine to multiple systems throughout the brain [98]. Though more conventionally associated with processes such as automatic arousal and cognitive flexibility, recent research has demonstrated that LC neurons play a pivotal, yet nuanced, role in modulating the circuitry underlying feeding. For instance, Yang et al., [2021] discovered that LC neurons co-released noradrenaline and glutamate to excite neurons in the parabrachial nucleus and suppress feeding in fear conditioned mice. Interestingly, LC neurons also suppressed inhibitory input to parabrachial nucleus (PBN) neurons by inducing the endocannabinoid-dependent and long-term depression of synapses in the CeA, whereas blocking or knocking out endocannabinoid receptors in CeA neurons prevented fear-induced suppression of feeding [99]. This suggests that antagonists of receptors on these LC pathways could potentially be used to modulate the impact of stress on eating.

Recent research has also shown that GABAergic cells of the LH can drive compulsive eating through direct projections to peri-LC neurons [100]. Contrary to literature implicating dopamine in food approach during physiologically satiated states [40], this study found that LH projections to the VTA were not implicated in induced eating, and that these fibers project through the VTA and synapse on a non-noradrenergic peri-LC target [100]. However, lesions to the peri-LC did not alter body weight, suggesting that this circuit may underlie the compulsive intake of food in absence of physiological hunger signals. This finding dovetails with research emphasizing the importance of peri-LC VGLUT2 neurons in modulating palatability and enhancing food consumption [101]. During food consumption, peri-LC VGLUT2 neuron inhibition response magnitude has been found to be scaled by palatability and homeostatic state. Correspondingly, peri-LC VGLUT2 neuron inhibition leads to increased food intake and enhanced palatability. These properties suggest that peri-LC neurons may comprise a double-negative feedback control mechanism that sustains food consumption without affecting food-seeking per se [102].

Efforts so far to characterize the LC function in humans using fMRI have been hampered by its small size and location near the fourth ventricle, though advancements in imaging techniques have substantial improvements in obtaining reliable estimates of LC activity [103]. Initial research suggests that a negative correlation between eating-related disinhibition and central norepinephrine transporter availability in the LC may be present in individuals with obesity, though this effect needs to be tested in larger samples [104].

## Basal ganglia

### Dorsal striatum

Accumulating evidence has highlighted the potential role of the striatum in modulating eating behaviors due to its involvement in the reward circuitry, salience, and motor processes [105]. Emerging literature has further suggested a ventral-dorsal functional division that may differentially underpin goal-directed and compulsive eating. The ventral striatum (VS), which includes the NAcc, has been consistently found to facilitate feeding via the modulation of the ‘liking’ aspects of reward processing [106]. In contrast, the dorsal striatum (DS), which encompasses the caudate and putamen, has been associated with compulsively driven (i.e., ‘wanting’) food intake [107, 108]. Compulsive overeating may be characterized by insensitivity to food-related reward value (either increased or decreased) or to the presence of aversive health and emotional outcomes [109–111]. As such, habitual overeating, especially in clinical populations, may be understood as the result of aberrant food-reward learning – a process potentially underpinned by the shift from VS- to DS-oriented response to food-related stimulus [107, 112]. For instance, increased activation in the VS and the DS in response to food images has been observed in women with binge eating disorder (BED) and bulimia nervosa, respectively [113, 114]. Similarly, a recent multimodal investigation reported resting-state hypoconnectivity between the ventral and dorsal caudate and frontal control regions (e.g., superior frontal gyrus) in adults with BED compared with healthy controls, and more widespread connectivity differences with the DS [115]. These findings were interpreted to reflect the dysfunctional integration of reward signals and self-regulation that contribute to binge eating.

Altered caudate reactivity to food-related stimuli (i.e., increased reactivity to food cue and attenuated reactivity to food receipt) has consistently been associated with risks of obesity, weight gain, and impulsivity [116–119]. Moreover, lower caudate and putamen resting-state functional connectivity has been found to correlate with greater impulsivity during food decision-making and longitudinal body mass index (BMI) gains [117]. This corresponds with findings identifying associations between heightened DS-somatosensory cortex functional connectivity with increased food craving and subsequent BMI gains [105]. More precisely, the posterior part of the dorsal putamen has been suggested to contribute to habitual eating via its role in taste and habit processing [108, 120]. Furthermore, caudate-precuneus and caudate-lateral PFC connectivity have been associated with inhibitory control, impulsivity relevant to weight management, and success in controlling weight [121, 122]. The adaptation in DS functioning has thus been interpreted to reflect altered reward processing and the formation of habitual eating that may inform our understanding of overeating.

Having said that, past studies have been limited by constraints in fMRI spatial resolution and may not be able to further explore the functional dissociation between the medial- and lateral-DS that may be relevant to the development of habit-based behaviors [105, 111, 112]. To date, rodent models have demonstrated that changes in activity of the medium spiny neurons of the dorsolateral striatum (DLS) reflect the process of performance optimization and action chunking as movements become repetitive and consistent [112]. Moreover, stress exposure has been shown to accelerate habit formation and to induce compulsive eating upon food withdrawal [111]. This acceleration may potentially be facilitated via the functional connection between the amygdala and the putamen, and the modulatory pathway from the CeA to the substantia nigra pars compacta, which supply dopaminergic input to the DLS [112]. Hence, investigation into the interaction between the striatum and the stress-response network may be a worthy pursuit for future studies on overeating. Future studies could explore how the CeA-putamen pathway may be involved in emotionally driven eating, and how this pathway may be modulated by cognitive control regions.

### Ventral pallidum and ventral striatum

The ventral pallidum (VP) is a main node in the basal ganglia, which receives strong GABAergic inhibitory input from the NAcc, comprising part of the VS, while also sending inhibitory signals to several regions associated with food intake, including the LH and VTA [123]. Animal research supports that the VP, alongside other regions such as the NAcc shell, the medial OFC, the posterior insula, and the PBN of the brainstem pons, serve as hedonic (‘liking’) hotspots in which local manipulations on opioid, orexin, endocannabinoid receptors lead to enhanced positive expressions to taste [18]. Within this context, the anterior insula plays a particularly important role in maintaining stable conceptual representations of food categories, whereas the caudal mid-insula supports flexibility depending on the current state of the body [124]. Subsequently, this highly processed visceral/autonomic taste, and olfaction information influences behavioral responses mediated by the VS [125]. Subpopulations of VP neurons have recently been found to inhibit NAcc neurons and increase ongoing food consumption during a free-access food paradigm while amplifying hedonic reactions to reward [126]. There is evidence to support the presence of an analogous system in humans, with fMRI studies demonstrating that activity in the VP and OFC are strongly modulated during moment-to-moment food pleasantness inferences [127]. Interestingly, another study using ratings of liking and wanting of food odors found evidence of higher activation in the VP for wanting than for liking during the hunger state when compared to a satiety state [128]. These results suggest a central role for the VP in the promotion of reward consumption through modulation of NAcc firing in a value-dependent, and possibly hunger-dependent, manner. Ultra-high field fMRI may be particularly well suited to model the dynamic interactions between the VP and NAcc during hungry and satiated states considering the obstacles in imaging small subcortical regions located near ventricles using standard field strengths [129].

The VP has also shown promise as a potential predictor for future weight gain. In one study, Burger and Stice [130] observed a simultaneous decrease in putamen and VP response during the receipt of a milkshake over repeated exposures, which was suggested to reflect food reward habitation. Next, cue–reward learning and habituation slopes were tested to predict weight gain over the course of two years and participants presenting the steepest escalation in VP response to food cues and the steepest reduction in caudate response to milkshake receipt had significantly larger increases in BMI. This was implied to suggest that increased dopamine signaling in response to predictive cues and decreased response to food receipt may constitute qualitatively divergent risk factors for future weight gain [130]. This finding complements prior research demonstrating a negative correlation between BMI and VP response during the reallocation of attention to unappetizing food images, thereby suggesting that VP response factors into food cues exerting a more powerful motivational effect in individuals with higher BMI [131]. Further research is needed to determine whether altered VP response represents a stable biomarker of excess weight gain or if alterations in VP activity are normalized following bariatric surgery.

## Recommendations and conclusions

**Fig. 1:**
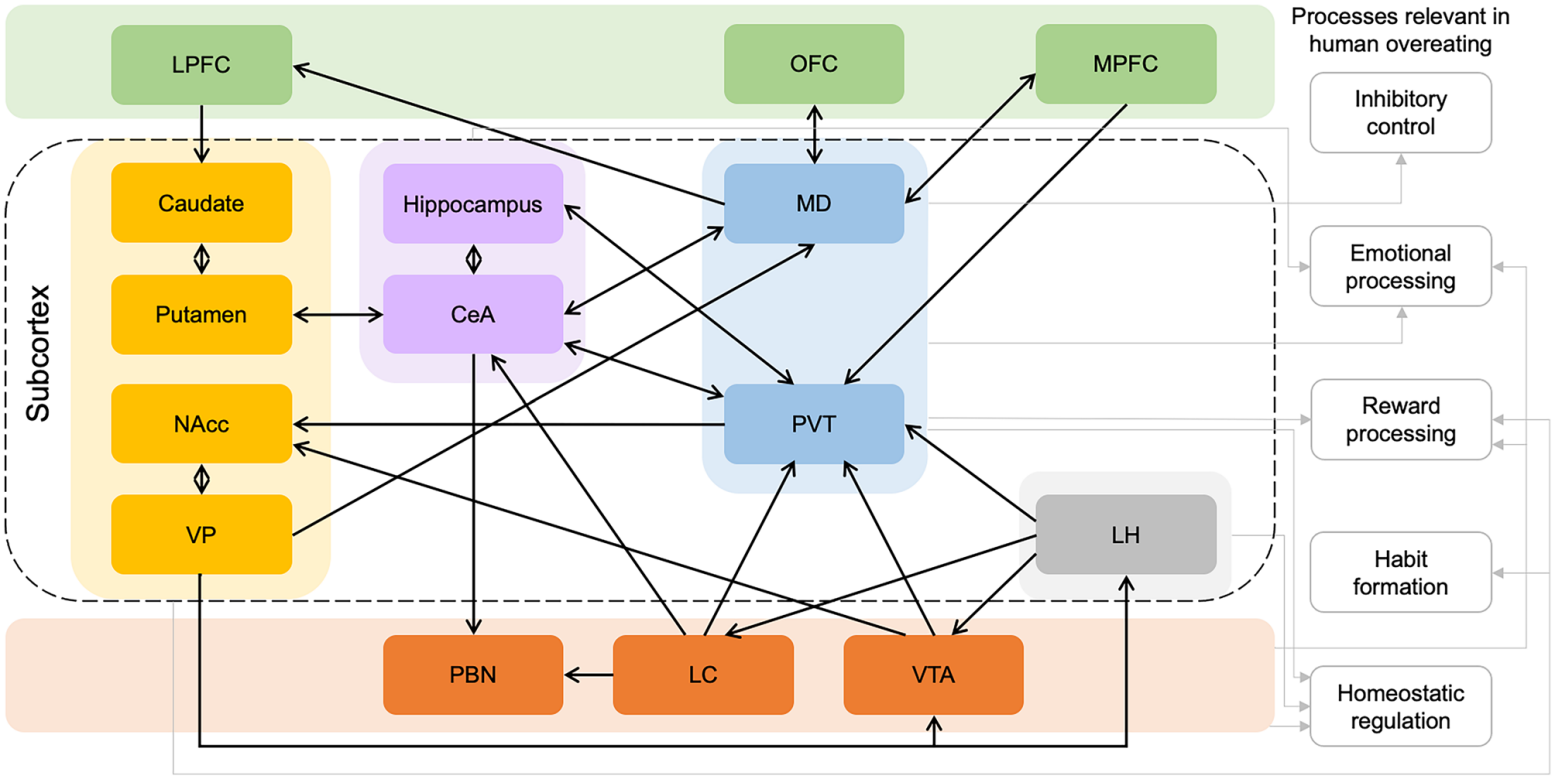
Subcortical connections involved in human overeating. Color-filled boxes represent the brain regions associated with human eating behaviors. Green-filled boxes represent prefrontal cortex regions, yellow-filled boxes represent striatal subdivisions, purple-filled boxes represent basal ganglion regions, blue-filled boxes represent thalamic nuclei, grey-filled boxes represent hypothalamic nuclei, and orange-filled boxes represent brainstem regions. The black arrows represent functional connections hypothesized to contribute to human overeating, the direction of which depicts input, output, or bidirectional pathways between regions. The grey-outlined boxes describe the key processes relevant to human overeating, and the grey arrows reflect the subcortical and brainstem regions associated with each of the processes. In this case, an expansive subcortical network is interpreted to integrate the homeostatic, reward learning, emotion processing, habit formation and cognitive control systems to dynamically influence the development and persistence of overeating. Abbreviations: LPFC, lateral prefrontal cortex; OFC, orbitofrontal cortex; MPFC, medial prefrontal cortex; NAcc, nucleus accumbens; VP, ventral pallidum; CeA, central nucleus of the amygdala; MD, mediodorsal thalamus; PVT, paraventricular thalamus; LH, lateral hypothalamus; PBN, parabrachial nucleus; LC, locus coeruleus; VTA, ventral tegmental area

As illustrated above, the potential of functional neuroimaging to advance our understanding of the subcortical and brainstem drivers of eating behavior is substantial because of its capacity to elucidate the neurobiological alterations underlying uniquely human overeating behaviors and provide insights into potential targets for focal non-invasive brain stimulation or pharmacological interventions [132, 133]. The subcortical and brainstem regions highlighted in this review tend to converge on demonstrating how pathways originating in the subcortex and brainstem can have a profound downstream impact on both homoeostatic signaling, as well as higher-order cognitive processes traditionally believed to be primarily mediated by the cortex (see Fig. 1). Overeating triggered by food cues appears contingent on the dynamic interplay between reward, motivational processing and whole-body energy regulation. The relevant neural signals originating from the brainstem (e.g., LC, VTA) and striatal subdivisions (e.g., NAcc, VP) converge in the intermediary thalamic nuclei (e.g., PVT) to facilitate the coordinated and flexible adjustment of food-approaching behaviors. Rather than being consciously motivated by the reward value of food, compulsive overeating may develop as ingestion becomes habitual or triggered by emotional and environmental cues. The transition from VS- to DS-oriented functioning and the interconnection between DS regions (e.g., caudate, putamen) and the emotion/stress response centers (e.g., amygdala) may contribute to overeating that is insensitive to the outcome value and instead propelled by stress-related processes. Aberrant functioning of corticothalamic circuits, of which the MD plays a critical role, may also potentially contribute to the cognitive control defects seen in populations that overeat. Other subcortical and brainstem regions known to contribute to ingestive behaviors and taste perception which were not extensively covered in this review include the solitary nucleus and the ventral posteromedial nucleus of the thalamus [134]. Notably, the lateral habenula (LHb) has received increasing attention, with accumulating evidence highlighting its role in negative reward signaling and psychiatric disorders associated with dysregulated reward processing [135, 136]. Rodent studies have reported that the inhibition of the LH-LHb glutamatergic projections increased consumption of palatable liquid in mice, while activation of the glutamatergic neurons in the VP excited the habenula-tegmental circuitry to indirectly decrease VTA dopaminergic neuron firing, resulting in constrained reward seeking [137, 138]. Additionally, resting-state functional connectivity between the LHb and the LC was reported to positively correlate with suicidal ideation in patients with past anorexia nervosa, highlighting the potential clinical relevance of neural adaptations in the LHb in eating disorders [139]. Further research is needed to determine the impact of the overconsumption of processed foods containing high amounts of sugars and fats on taste perception and whether the modulation of post-ingestive nutritional reward value alters pathways controlling feeding in a manner that can lead to overeating.

### The untapped potential of ultra-high field MRI

**Fig. 2:**
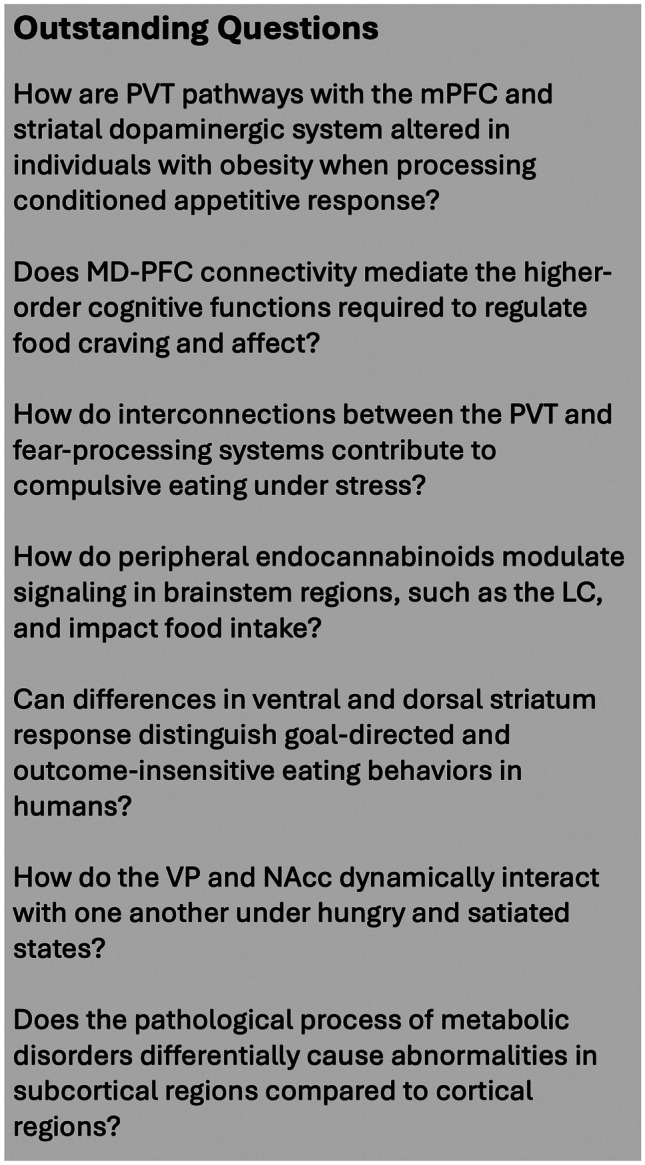
Outstanding questions on how subcortical and brainstem regions contribute to overeating

Image acquisition and functional analysis of the human subcortex and brainstem using fMRI are particularly challenging due to the small size, complex interconnectivity, and propensity to physiological noise of these brain regions. Nevertheless, the broader adoption of ultra-high field 7 T MRI scanners and other methodological advances to correct for physiological noise have facilitated the imaging of brain regions beyond the cortex [140, 141]. For example, brainstem nuclei often have an average cross-sectional diameter of only a few millimeters, which is problematic considering that fMRI at field strengths of 3-Tesla and below provides an in-plane spatial resolution of approximately 2–4 mm and customarily undergo smoothing with kernels between 5–8 mm [142]. Recent research has shown that fMRI at 7 T can overcome these fundamental acquisition challenges by using unsmoothed single-subject data to differentiate brain activation and provide highly sensitive connectivity parameters in substructures characterized by signal dropouts [143]. Moreover, the increased temporal resolution of 7 T fMRI has been harnessed to map the complex topographic organization of the subcortex by means of large-scale functional connectivity gradients [144]. Such tools hold promise for establishing reproducible brain–behavior relationships and pinpointing the neural pathways that contribute to maladaptive eating behaviors on an individual level. Relatedly, multi-echo fMRI has also been demonstrated to substantially improve the reliability of functional connectivity-based measurements [145, 146], and to increase temporal signal-to-noise ratios (tSNR) at 7 T [147]. Figure 2 provides a list of outstanding questions on how subcortical and brain stem function contributes to overeating that could possibly be addressed with functional neuroimaging.

### Specific considerations for examining eating behaviors with neuroimaging

Although neuroimaging has tremendous potential impact to inform fields ranging from psychiatry, neuroscience, to nutrition, the proper utilization of neuroimaging techniques and suitable caution when interpretating findings is required [148]. Providing detailed guidelines on how to appropriately conduct neuroimaging studies examining eating behaviors lies outside the purview of this review, though we highly suggest consulting reviews on good practice in food-related [149] and eating disorder [150] neuroimaging before designing a study to address a specific research question. These guidelines provide recommendations on how to address considerations specific to eating behavior, such as hunger state, task design, data quality control procedures, menstrual phase, and BMI. Taking into consideration recent, and alarming, research showing that common task-fMRI measures are not currently suitable for brain biomarker discovery or for individual-differences research, it is imperative that efforts be made to improve task-fMRI reliability [151, 152]. Relatedly, issues that are still rampant in neuroimaging research, such as low statistical power, researcher degrees of freedom in data analysis, and lack of direct replication can now be more easily addressed by virtue of the multitude of open-source tools to aid researchers in adhering to proposed best practices [153, 154]. Such design considerations and data-sharing initiatives represent an opportunity to transform the role of neuroimaging in understanding the neurobiological mechanisms of overeating and in developing clinical applications for brain-derived measures [155, 156].

## Abbreviations

7T: 7-Tesla
BED: Binge eating disorder
BOLD: Blood oxygen level dependent
BMI: Body mass index
CeA: Central nucleus of the amygdala
DLS: Dorsolateral striatum
DS: Dorsal striatum
fMRI: Functional magnetic resonance imaging
LC: Locus coeruleus
LH: Lateral hypothalamus
LHb: Lateral habenula
LPFC: Lateral prefrontal cortex
MD: Mediodorsal thalamus
MPFC: Medial prefrontal cortex
NAcc: Nucleus accumbens
OFC: Orbitofrontal cortex
PBN: Parabrachial nucleus
PVT: Paraventricular thalamus
SMS: Simultaneous multi-slice
tSNR: Temporal signal-to-noise ratio
VP: Ventral pallidum
VS: Ventral striatum
VTA: Ventral tegmental area
